# Preparation, characterization, and application of camellianin A/soy protein isolate covalent complexes

**DOI:** 10.3389/fnut.2025.1639554

**Published:** 2025-07-28

**Authors:** Peng Gao, Yuanyang Nie, Yingxuan Zhou, Wupeng Ge

**Affiliations:** ^1^College of Food Science and Engineering, Northwest A&F University, Yangling, China; ^2^School of Food Science, Henan Institute of Science and Technology, Xinxiang, China

**Keywords:** camellianin A, soy protein isolate, covalent complex, nanoemulsion, characterization

## Abstract

Alkaline-assisted processing facilitated the development of camellianin A (CA)-soy protein isolate (SPI) conjugates, with systematic characterization of polyphenol incorporation effects on macromolecular architecture and emulsion stabilization capacity. Multi-spectroscopic profiling confirmed CA-induced structural reorganization of SPI matrices, verifying conjugate formation. Dose-dependent enhancement patterns were observed, where increased CA loading positively correlated with total phenolic content, surface hydrophilicity, ABTS radical scavenging activity and reducing power. The derived nanoemulsions demonstrated superior oxidative stabilization capacity and storage stability, outperforming SPI-only stabilized systems. Our results can advance the utilization of CA in functional foods, providing practical guidance for developing novel food-grade nanoemulsions with enhanced delivery efficacy.

## 1 Introduction

Interactions between proteins and polyphenols are inevitable in food matrices, leading to modifications in their structural configurations, functional properties, and physicochemical characteristics, which subsequently influence food flavor, color, and nutritional value (1, 2). These interactions primarily occur through two distinct mechanisms: covalent and non-covalent binding. Non-covalent associations, mediated by electrostatic forces, hydrophobic interactions, hydrogen bonding, electrostatic forces, and van der Waals attractions, are generally reversible and susceptible to environmental changes (3). In contrast, covalent conjugation between polyphenols and proteins forms stable polymeric complexes that can significantly enhance protein functionality, including improved emulsifying capacity, increased antioxidant activity, and reduced allergenicity under specific conditions (4, 5). Notably, Liu et al. (6) demonstrated that radical-induced coupling of β-lactoglobulin with chlorogenic acid enhanced both thermal stability and antioxidant properties of the protein. Covalent conjugation has been widely employed in functional food development for encapsulating and controlling the release of bioactive compounds, thereby improving their bioavailability and stability (7, 8). For instance, Gu et al. (9) prepared ovalbumin-catechin conjugates via free radical grafting, revealing that polyphenol incorporation not only augmented the protein’s antioxidant capacity but also effectively suppressed β-carotene oxidation while enhancing its biological utilization.

Soy protein isolate (SPI), containing all essential amino acids, is widely utilized in food industry due to its excellent functional properties including superior emulsifying capacity and gelation ability (10). However, its susceptibility to denaturation under extreme pH, high ionic strength, or elevated temperatures often compromises its functionality, thereby limiting applications in complex food systems (11). Camellianin A (CA), the predominant flavonoid (>20% content) in *Adinandra nitida* leaves, possesses remarkable bioactivities (12). Liu et al. (13) demonstrated its potent antioxidant capacity, while Gao et al. (14) reported significant inhibitory effects on HepG2 and MCF-7 cancer cell proliferation. Jia et al. (15) further identified its *α*-glucosidase inhibitory activity. Although Zhou et al. (16) investigated non-covalent CA-lysozyme complexes for nanoemulsion stabilization, covalent conjugation between CA and food-grade proteins remains unexplored. This study pioneers the covalent conjugation of CA with SPI, systematically characterizing the structural modifications and functional enhancements of the resultant complexes. The research specifically evaluates their application in nanoemulsions as novel emulsifiers.

## 2 Materials and methods

### 2.1 Chemicals

CA was from our previous report (16). Soy protein isolate (SPI) was provided by Mantianxue Food Manufacturing Co., Ltd. (Anyang, China). The sunflower oil was the product of COFCO Fulinmen Food Marketing Co., Ltd. (Beijing, China). Medium-chain triglycerides (MCT), 8-Anilino-1-naphthalenesulfonic acid (ANS), 2,2′-Azino-bis(3-ethylbenzothiazoline-6-sulfonic acid) diammonium salt (ABTS), and Folin–Ciocalteu’s phenol reagent were purchased from Yuanye Biological Technology Co., Ltd. (Shanghai, China). All other reagents were of analytical grade.

### 2.2 Preparation of CA/SPI covalent complex

The CA/SPI covalent complexes were prepared through an alkaline-induced method (17). Specifically, SPI was dissolved in 100 mL deionized water and adjusted to pH 9.0 using 0.5 mol/L NaOH solution to obtain a 0.5 wt% protein solution. The solution was allowed to swell overnight at 4°C to ensure complete hydration, with sodium azide (0.005%) added as a microbial inhibitor. For covalent conjugation, 100 mL of CA solution at varying concentrations (0.02 wt%, 0.05 wt%, and 0.1 wt%) was gradually added to 100 mL of the protein solution under constant stirring. The reaction mixture was readjusted to pH 9.0 with 0.5 mol/L NaOH and maintained for 24 h. Subsequently, the resulting complexes were dialyzed (molecular weight cutoff: 3,500 Da) for 48 h and lyophilized to obtain the final products, designated as CA/SPI-1, CA/SPI-2, and CA/SPI-3 corresponding to the increasing CA concentrations.

### 2.3 UV and FT-IR spectra

The UV spectra (220–400 nm) of CA, SPI, and CA/SPI covalent complexes were acquired using a TU-1810 spectrophotometer (Persee Analytical, China). Fourier-transform infrared (FT-IR) spectra (4000 to 400 cm^−1^) was collected on a Bruker INVENIO spectrometer (Bruker Optics, Germany).

### 2.4 Determination of total phenolic content

The total phenolic contents (TPC) of the three CA/SPI covalent complexes were analyzed based on the Folin–Ciocalteu method (18). Sample solutions were prepared at a concentration of 5 mg/mL. For analysis, 0.3 mL of each sample solution was diluted to 10 mL with distilled water, followed by addition of 0.5 mL Folin–Ciocalteu reagent. After vortexing for 5 min, 5 mL of Na_2_CO_3_ (5% w/v) was added, and the final volume was adjusted to 25 mL with H_2_O. The reaction mixtures were incubated in the dark for 90 min before measuring absorbance at 750 nm against an ultrapure water blank. TPC was calculated based on a standard curve prepared with CA and expressed as mg CA equivalents per g sample (mg CAE/g).

### 2.5 Fluorescence spectra

Sample solutions of SPI, CA/SPI-1, CA/SPI-2, and CA/SPI-3 were prepared at a concentration of 1 mg/mL using 10 mmol/L PBS buffer (pH 7.0). Fluorescence emission spectra (300–450 nm) at *λ*_ex_ = 280 was collected on a fluorescence spectrophotometer.

### 2.6 Surface hydrophobicity measurement

The surface hydrophobicity values of SPI, CA/SPI-1, CA/SPI-2, and CA/SPI-3 were evaluated using ANS as a fluorescent probe (19). SPI and three CA/SPI covalent complexes were diluted to concentrations of 0.05–0.30 mg/mL with 10 mmol/L PBS (pH 6.5). For each measurement, 20 μL of ANS solution (8 × 10^−3^ mol/L) was added to 4 mL of protein solution and mixed thoroughly. After 10 min of incubation in the dark, fluorescence emission spectra (400–700 nm) were recorded at *λ*_ex_ = 390 nm using a fluorescence spectrophotometer. The maximum fluorescence intensity was plotted against protein concentration, with the slope of the resulting linear regression representing the relative surface hydrophobicity index (H_0_).

### 2.7 Determination of ABTS radical scavenging activity

The ABTS radical scavenging assay of CA, SPI, and CA/SPI covalent complexes was carried out based on the report of Gu et al. (20). The ABTS test solution was prepared by mixing 200 mL of 7 mmol/L ABTS stock solution with 3.52 mL of 2.45 mmol/L potassium persulfate solution, followed by 12 h incubation in the dark at room temperature. The resulting solution was diluted with 0.05 mol/L PBS (pH 7.4) to achieve an absorbance of 0.70 ± 0.02 at 734 nm after 30 min equilibration. For the assay, 0.15 mL of sample solution at various concentrations was mixed with 2.85 mL of the diluted ABTS radical test solution. After vortexing and 10 min reaction in the dark at room temperature, the absorbance (A_t_) was measured at 734 nm using a spectrophotometer. A control measurement (A_0_) was performed using 0.15 mL deionized water instead of sample solution. The ABTS radical scavenging activity was calculated as Equation 1:


(1) $$\text{ABTS scavenging activity}\;(\%)=[1-(A_{t}/A_{0})]\times 100$$

### 2.8 Reducing power assay

The reducing power of CA, SPI, and CA/SPI covalent complexes was evaluated following the method established by Du et al. (21). In brief, 0.5 mL of sample solution at various concentrations was mixed with 2.5 mL of 0.2 mol/L PBS (pH 6.6) and 2.5 mL of 1% potassium ferricyanide solution. The mixture was vortexed thoroughly and incubated at 50°C for 20 min. After rapid cooling in an ice-water bath, 2.5 mL of 10% trichloroacetic acid solution was added, followed by centrifugation at 4,500 rpm for 10 min. Subsequently, 2.5 mL of the supernatant was combined with 2.5 mL ultrapure water and 0.5 mL of 0.1% ferric chloride solution. The reaction mixture was kept at room temperature for 10 min before measuring the absorbance at 700 nm using a spectrophotometer. The reducing power was expressed as the absorbance at 700 nm.

### 2.9 Preparation of nanoemulsions of CA/SPI covalent complexes

The nanoemulsions were prepared based on a previous report (22). Briefly, SPI and three CA/SPI covalent complexes (CA/SPI-1, CA/SPI-2, CA/SPI-3) were individually dissolved in ultrapure water at 4% (w/v) concentration to form the aqueous phase, while MCT served as the oil phase. The two phases were mixed at a 95:5 (v/v) ratio and homogenized at 15,000 rpm for 2 min to obtain coarse emulsions. For nanoemulsification, aliquots of the coarse emulsion were subjected to ultrasonic treatment using a probe sonicator under the following optimized parameters: 5 min total processing time at 660 W power output, with 2 s pulse-on and 2 s pulse-off intervals. The temperature was maintained below 25°C throughout sonication by employing an ice-water bath.

### 2.10 Storage stability evaluation of nanoemulsions

The storage stability of nanoemulsions prepared with SPI, CA/SPI-1, CA/SPI-2, and CA/SPI-3 was systematically evaluated under two temperature conditions (4°C and 25°C). At predetermined intervals, the emulsions were characterized for droplet size distribution using a Nano-ZS dynamic light scattering analyzer (Malvern Instruments, United Kingdom) (23). Prior to analysis, samples were diluted 1,000-fold with ultrapure water to minimize multiple scattering effects.

### 2.11 Evaluation of lipid oxidation resistance

Nanoemulsions containing SPI, CA/SPI-1, CA/SPI-2, and CA/SPI-3 were prepared according to Section 2.9, using sunflower oil as the oil phase. The emulsions were stored at 37°C to accelerate oxidation, with periodic determination of peroxide values (PV) (24). Briefly, 200 μL of emulsion was mixed with 1.5 mL of isooctane/2-propanol (3:1, v/v) in a 10 mL centrifuge tube through three cycles of 10 s vortex mixing. After centrifugation at 2,000 × g for 5 min and 3 min standing, 200 μL of the upper organic phase was transferred to a test tube containing 2.8 mL of methanol/1-butanol (2:1, v/v), followed by sequential addition of 15 μL ammonium thiocyanate solution (3.94 M) and 15 μL ferrous ion solution. The mixture was vortexed and incubated at room temperature for 20 min before measuring absorbance at 510 nm against a blank consisting of pure sunflower oil. Hydrogen peroxide concentration was determined from a standard calibration curve.

### 2.12 Statistical analysis

Experimental results are presented as mean value ± standard deviation (*n* = 3). Statistical analysis were performed using SPSS 26.0 (IBM Corp., United States), including ANOVA, Pearson correlation analysis, and Duncan’s multiple comparison (significance level set at *p* < 0.05). Data visualization was conducted using Origin 8.0 (OriginLab Corp., United States).

## 3 Results and discussion

### 3.1 UV analysis

**Figure 1:**
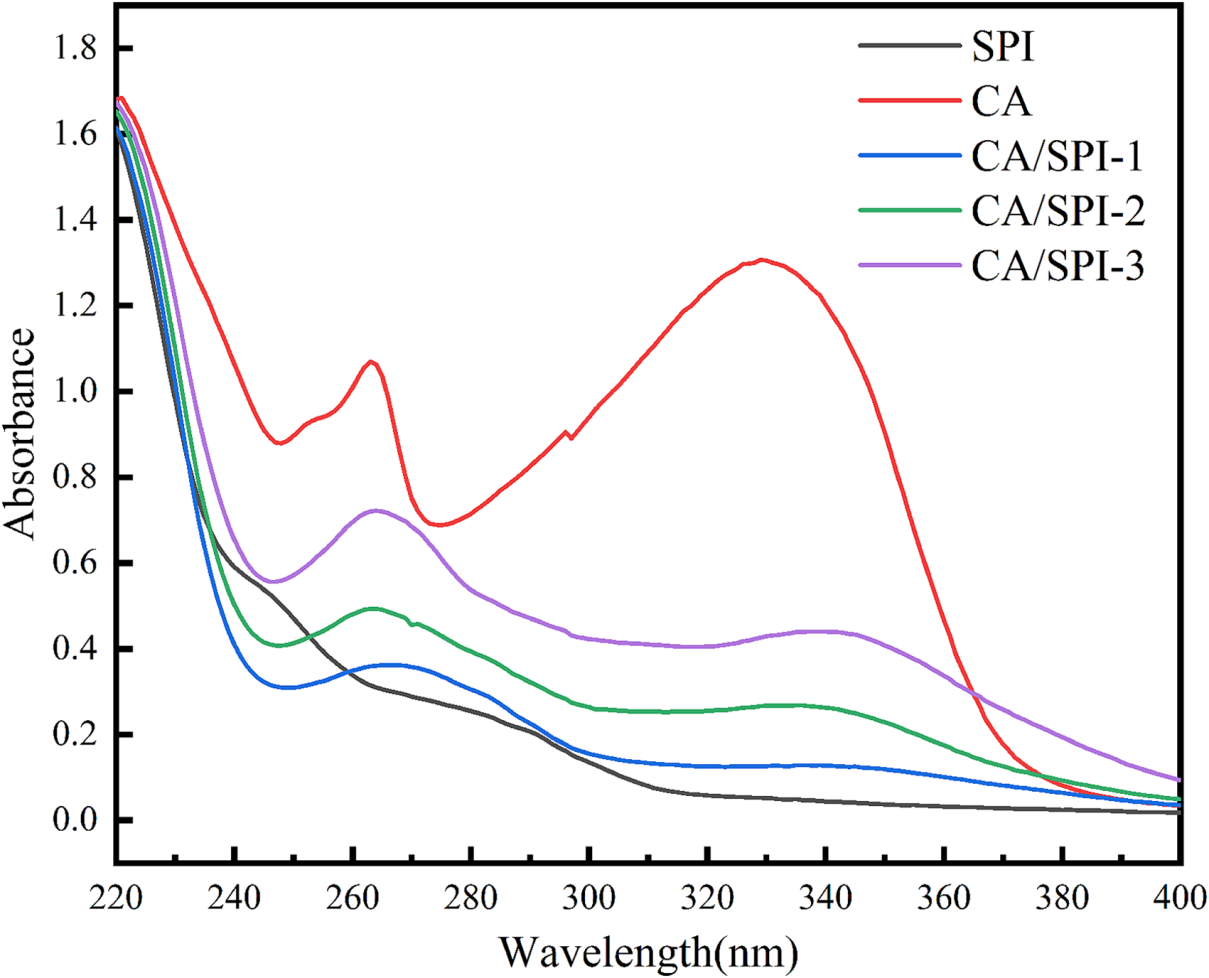
UV spectra of CA, SPI and their covalent complexes.

UV spectroscopy is widely employed to investigate interactions between proteins and polyphenols. This technique is particularly valuable because aromatic amino acid residues (e.g., tyrosine, phenylalanine, tryptophan) and sulfur-containing amino acids in proteins exhibit characteristic UV absorption (25). Changes in absorption peak positions and intensity can indicate molecular interactions between proteins and polyphenols. As shown in Figure 1, increasing the concentration of CA during preparation led to significant alterations in both the intensity and position of UV absorption peaks. Compared to pure SPI, the covalent complexes exhibited a blue shift of 2–10 nm in their absorption maxima. This spectral shift provides direct evidence of intermolecular interactions between CA and SPI, resulting in the formation of new chemical structures with modified electronic transitions.

### 3.2 FT-IR analysis

**Figure 2:**
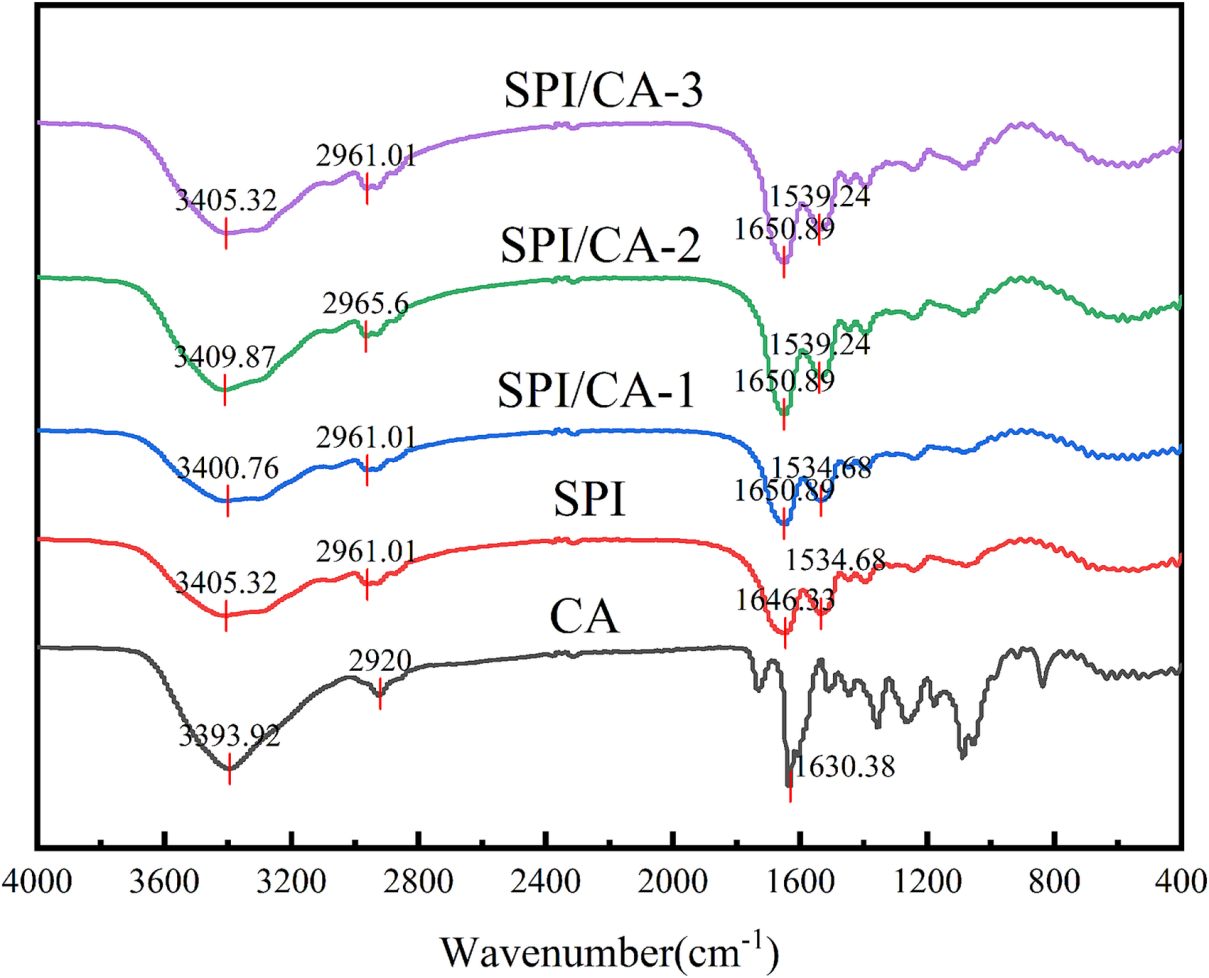
FT-IR spectra of CA, SPI and their covalent complexes.

FT-IR can characterize the structural modifications in CA, SPI, and their covalent complexes (Figure 2). The spectrum of CA displayed characteristic absorption bands at 3393.92 cm^−1^ (O–H stretching), 2,920 cm^−1^ (C–H stretching), and 1630.38 cm^−1^ (C=O stretching). For SPI, the absorption peaks appeared at 3405.32 cm^−1^ (O–H/N–H stretching), 2961.01 cm^−1^ (C–H stretching), 1660.89 cm^−1^ (C=O stretching), and 1534.68 cm^−1^ (C–N stretching/N–H bending). The covalent complexes (CA/SPI-1, CA/SPI-2, and CA/SPI-3) exhibited absorption bands at 3400.76–3409.87 cm^−1^, showing slight shifts from the original O–H/N–H vibrations of SPI and CA, suggesting potential hydrogen bond formation or covalent linkage through hydroxyl/amino groups. The persistent peaks at 2961.01–2965.6 cm^−1^ confirmed the preservation of aliphatic structures in the complexes. The amide I band shifted to 1650.89 cm^−1^, indicating alterations in the C=O vibrational environment, while the amide II bands at 1534.68–1539.24 cm^−1^ demonstrated modified N–H/C–N vibrations. The overall spectral patterns predominantly reflected SPI’s characteristic peaks, confirming its structural dominance in the complexes.

### 3.3 Total phenolic content

In this study, the Folin–Ciocalteu method was applied to quantify the content of CA covalently conjugated with SPI. The alkali-induced synthesis yielded CA/SPI covalent complexes with polyphenol contents of 126.65 ± 2.17 mg/g, 148.72 ± 2.84 mg/g, and 165.59 ± 6.66 mg/g, respectively. The results indicated a positive correlation between the incorporated total phenolic content and the increasing CA addition levels. Structural analysis revealed that the phenolic hydroxyl groups in CA could effectively graft onto nucleophilic groups (e.g., amino and sulfhydryl groups) of SPI through the oxidized quinone pathway, ultimately forming CA/SPI covalent complexes.

### 3.4 Fluorescence analysis

**Figure 3:**
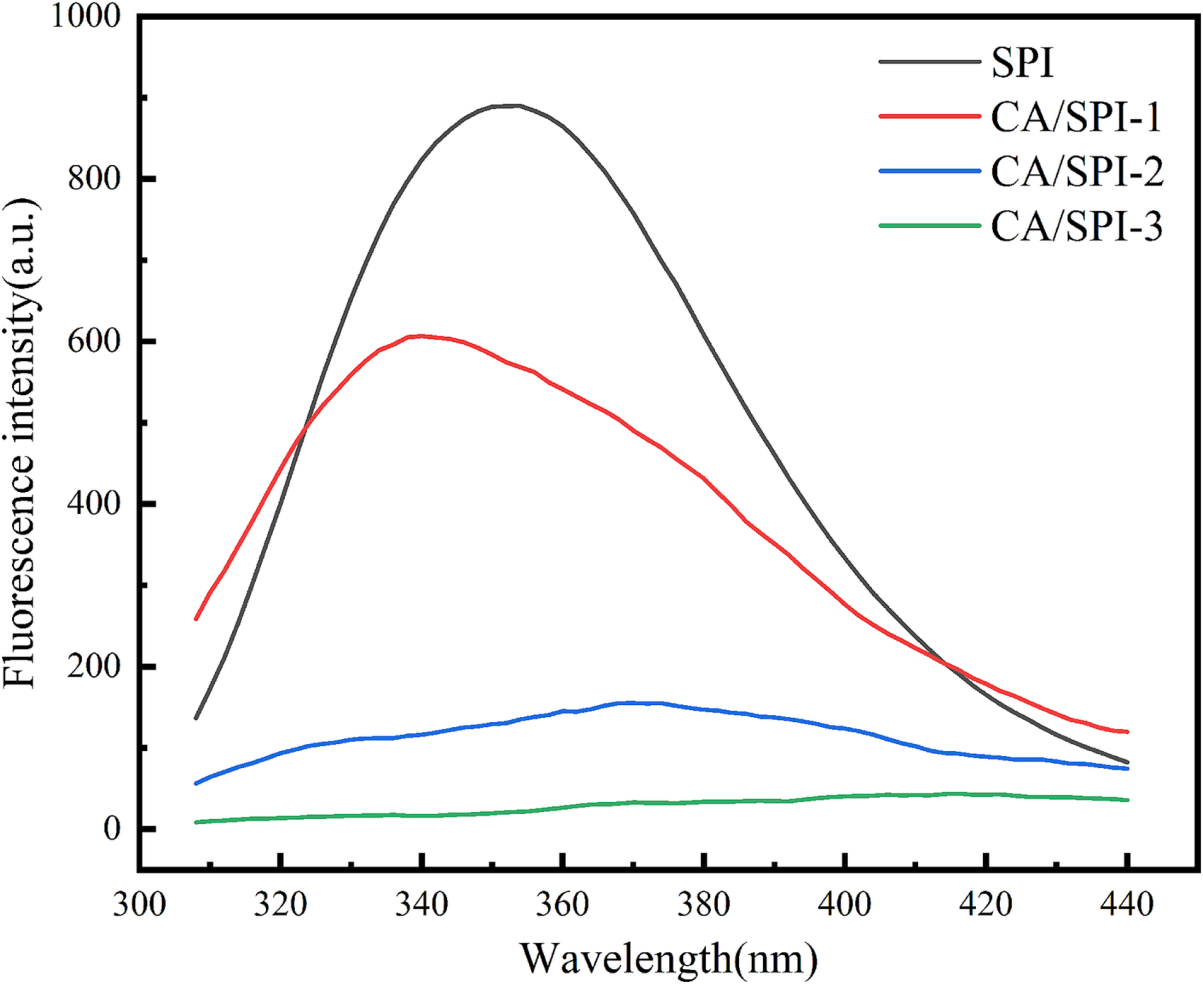
Fluorescence spectra of SPI and CA/SPI covalent complexes.

The aromatic amino acids in proteins, including tryptophan (Trp), tyrosine (Tyr), and phenylalanine (Phe), exhibit intrinsic fluorescence under specific excitation wavelengths due to their conjugated double-bond systems (26). These residues emit light at longer wavelengths than the incident radiation within nanoseconds. Alterations in the microenvironment of these fluorophores can be monitored through fluorescence spectral changes, thereby enabling structural characterization of protein-polyphenol interactions (27). In Figure 3, SPI displayed a characteristic fluorescence emission peak near 340 nm, consistent with typical protein fluorescence profiles. Notably, the CA/SPI complex demonstrated significant fluorescence intensity reduction compared to native SPI, indicating polyphenol-induced fluorescence quenching. This phenomenon may arise from structural proximity between CA and SPI’s fluorophores (e.g., Trp residues), where molecular interactions modulate excited-state energy dissipation.

### 3.5 Surface hydrophobicity analysis

**Figure 4:**
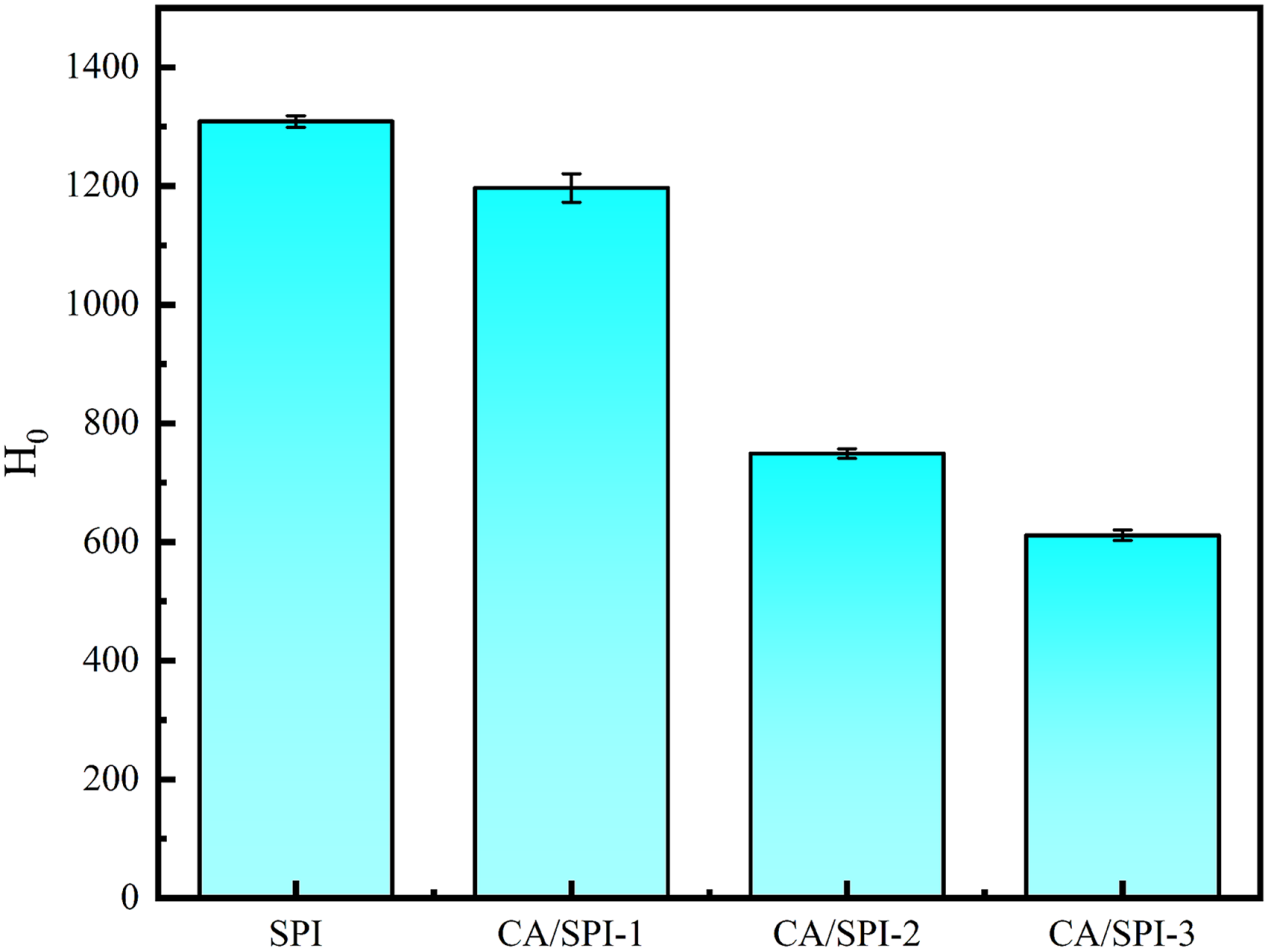
Surface hydrophobicity analysis of SPI and CA/SPI covalent complexes.

The surface hydrophobicity of proteins serves as a critical indicator for monitoring structural modifications (28). In this study, ANS was selected as the fluorescent probe to detect alterations in nanoparticle surface hydrophobicity. Surface hydrophobicity reflects the distribution pattern of hydrophobic amino acid residues on protein surfaces, where higher values correspond to greater exposure of these hydrophobic moieties (29). In Figure 4, SPI exhibited progressively diminished surface hydrophobicity with increasing CA concentrations. The fluorescence attenuation percentages followed this descending order: CA/SPI-3 (53.28%) > CA/SPI-2 (42.76%) > CA/SPI-1 (8.58%). This phenomenon may be associated with (a) molecular interactions between CA and hydrophobic domains of SPI, and/or (b) structural reorganization of the protein that modulates the spatial distribution of hydrophobic regions in aqueous systems.

### 3.6 ABTS radical scavenging activity

**Figure 5:**
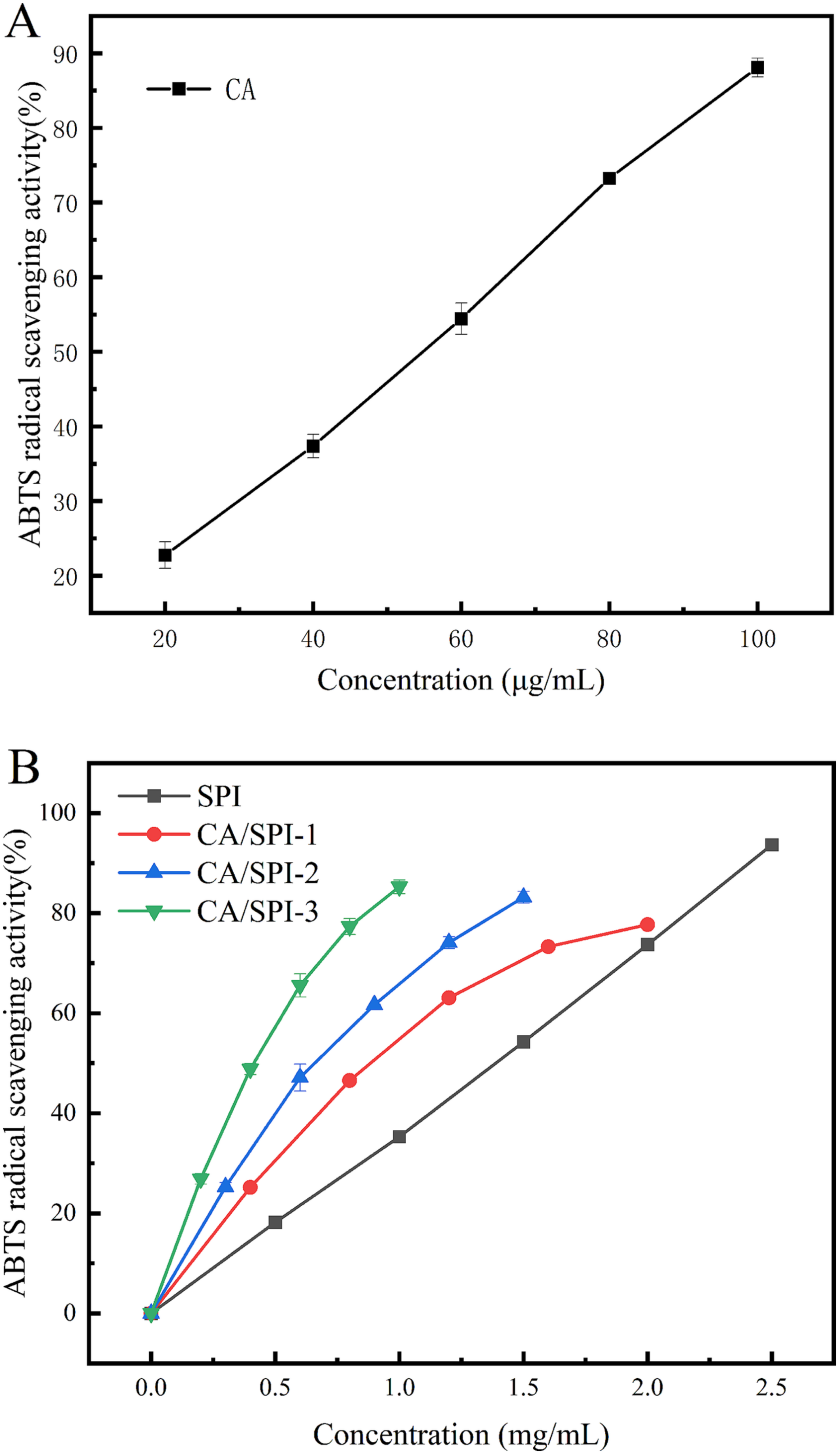
ABTS radical scavenging abilities of CA **(A)**, SPI and their CA/SPI **(B)** covalent complexes.

The ABTS test represents a widely adopted method for evaluating antioxidant capacity. Oxidized ABTS forms a stable bluish-green cationic radical (ABTS) exhibiting maximum absorbance at 734 nm (30). The scavenging rate against ABTS radicals quantitatively reflects antioxidant potency, with higher values indicating superior activity. As presented in Table 1 and Figure 5, CA, SPI, and their complexes demonstrated concentration-dependent radical scavenging capacities positively correlated with total phenolic content. The calculated IC_50_ values of CA/SPI complexes were significantly lower than those of SPI alone, with antioxidant enhancements reaching 29.14, 48.96, and 67.54%, respectively. These findings suggest structural integration between CA and SPI synergistically amplifies antioxidant performance. Such enhancement may be associated with redox-active moieties in the composite system, particularly hydroxyl-containing structures known to facilitate electron transfer processes (31).

**Table 1:** ABTS radical scavenging abilities of CA, SPI and CA/SPI covalent complexes.

Sample	IC_50_ (μg/mL)
CA	46.50 ± 0.46^e^
SPI	1193.29 ± 4.94^a^
CA/SPI-1	845.61 ± 5.95^b^
CA/SPI-2	609.05 ± 8.02^c^
CA/SPI-3	387.37 ± 1.13^d^

^a-e^ Subsequently, the resulting complexes were dialyzed (molecular weight cutoff: 3,500 Da) for 48 h and lyophilized to obtain the final products, designated as CA/SPI-1, CA/SPI-2, and CA/SPI-3 corresponding to the increasing CA concentrations.

### 3.7 Reducing power analysis

**Figure 6:**
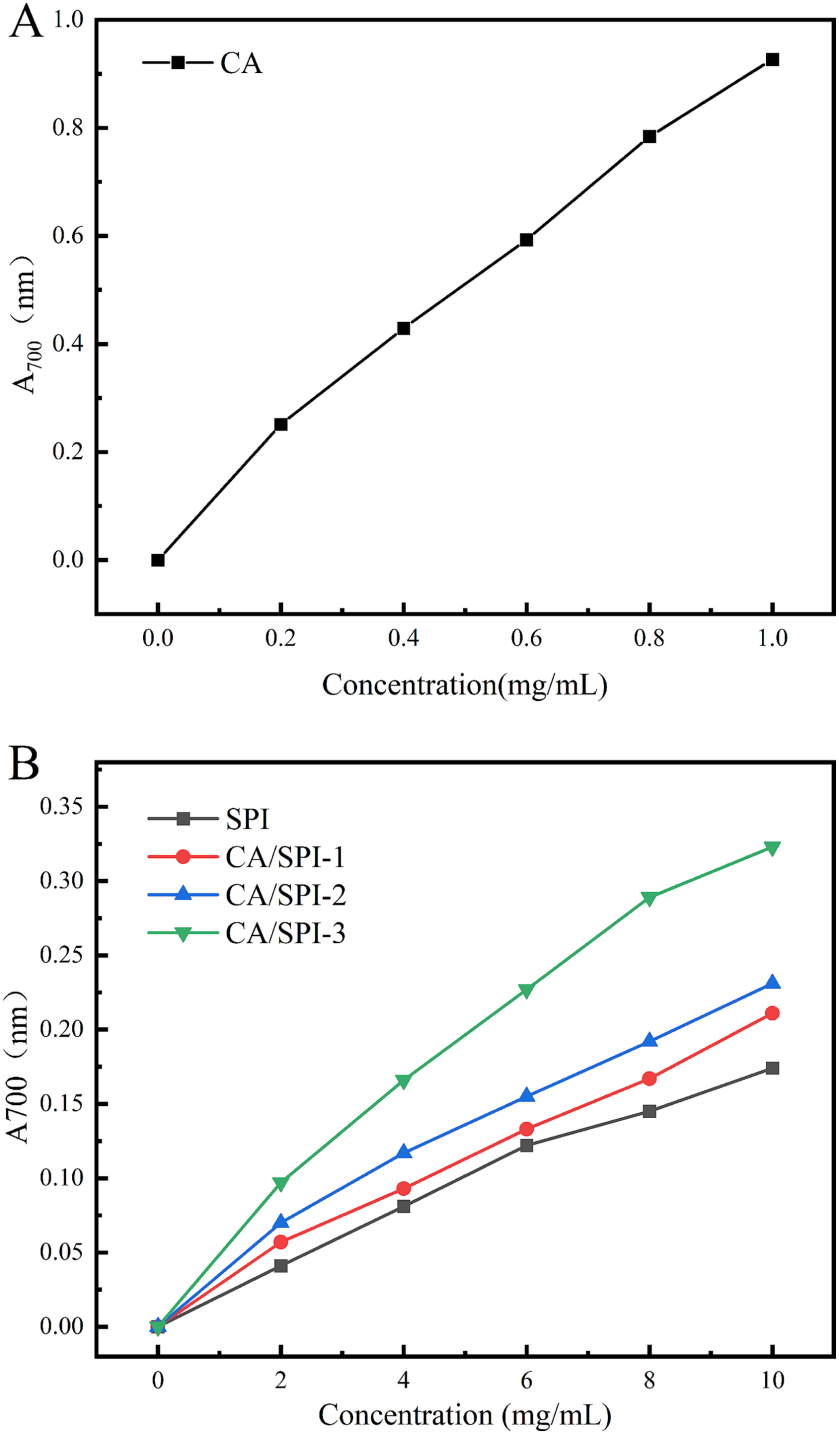
Reducing power analysis of CA **(A)**, SPI and their CA/SPI **(B)** covalent complexes.

The iron-reducing capacity reflects a substance’s ability to reduce Fe^3+^ to Fe^2+^, serving as a critical indicator of antioxidant potential. Protein-polyphenol complexes can mediate the reduction of potassium ferricyanide [K_3_Fe(CN)_5_] to ferrous ions, subsequently reacting with FeCl_3_ to form Prussian blue (Fe_4_[Fe(CN)_6_]_3_), which exhibits a characteristic absorption peak at 700 nm. The absorbance intensity positively correlates with reducing capacity (32). As illustrated in Figure 6, CA demonstrated the highest reducing capacity, followed by CA/SPI complexes. At 10.0 mg/mL concentration, the CA/SPI-1, CA/SPI-2, and CA/SPI-3 complexes exhibited 1.21-, 1.32-, and 1.86-fold higher reducing capacities than native SPI, respectively, showing positive correlation with total phenolic content. These findings align with the report of Yi et al. (33) on catechin-α-lactalbumin systems, where phenolic conjugation significantly enhanced reducing performance. Collectively, the data suggest that polyphenol-mediated protein modification effectively amplifies iron-reducing capabilities in composite systems.

### 3.8 Storage stability analysis

**Figure 7:**
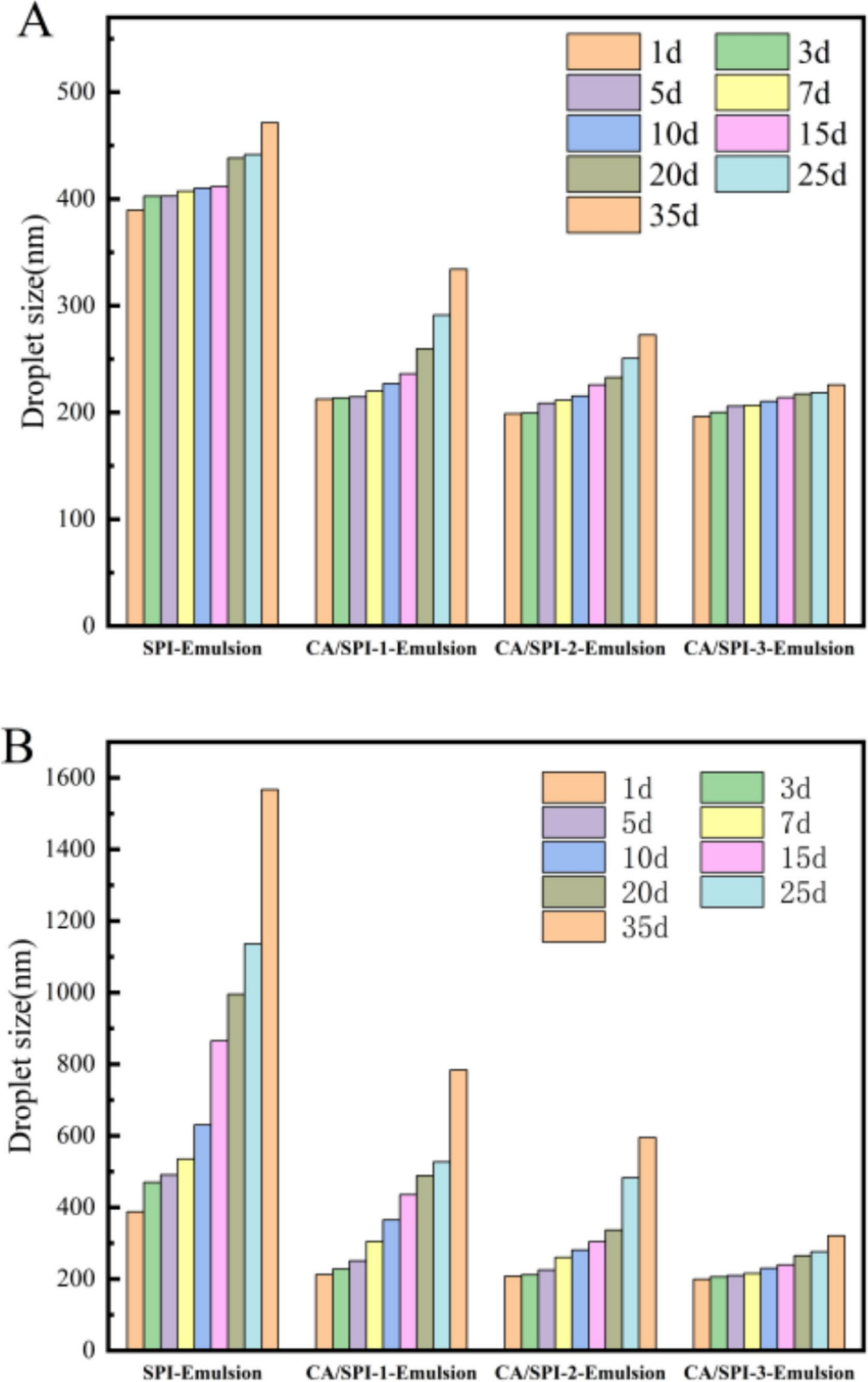
Changes in particle size of the nanoemulsions during storage at 4°C **(A)** and 25°C **(B)**.

Physical stability during processing, transportation, and storage constitutes a critical parameter for emulsion applications. Temperature variations critically influence emulsion destabilization patterns, with droplet size serving as a key indicator of droplet aggregation status (34). Smaller initial droplet diameters (<300 nm) typically correlate with enhanced stability against phase separation. Systematic monitoring of four emulsion systems during 35-day storage at 4°C and 25°C revealed distinct stabilization profiles (Figure 7). Freshly prepared SPI-Emulsion exhibited an initial particle size of ~390 nm, while CA/SPI-derived nanoemulsions maintained diameters around 210 nm. Storage-induced particle growth proved most pronounced in SPI-Emulsion, culminating in phase separation at 25°C after 35 days. In contrast, CA/SPI-3-Emulsion retained sub-330 nm diameters under both storage conditions, with significantly attenuated growth rates at 4°C versus 25°C. The observed thermal activation effects on particle aggregation suggest temperature-modulated molecular mobility influences destabilization kinetics. Improved stabilization in CA/SPI systems likely stems from reinforced interfacial architecture capable of counteracting thermodynamic destabilization drivers.

### 3.9 Anti-lipid oxidation analysis

**Figure 8:**
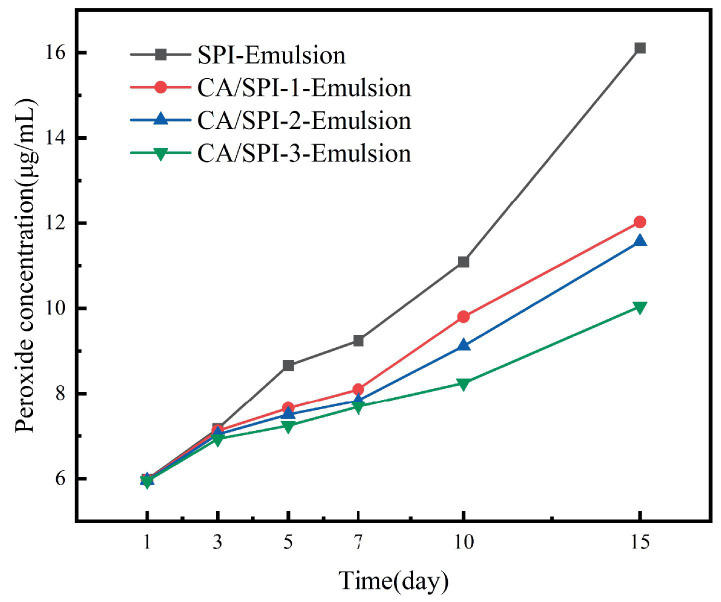
Peroxide content changes in nanoemulsions during storage.

The enhanced surface-area-to-volume ratio of nanoemulsions facilitates interfacial interactions between lipid components and pro-oxidants, potentially accelerating oxidation kinetics (35, 36). Sunflower oil-based systems, characterized by elevated unsaturated fatty acid content (predominantly oleic and linoleic acids), demonstrated progressive lipid oxidation during 15-day storage at 37°C. Peroxide value evolution, as shown in Figure 8, revealed oxidation inhibition capacities in the descending order: CA/SPI-3 > CA/SPI-2 > CA/SPI-1 > SPI, correlating with phenolic constituent levels. Santos et al. (37) also found that the lipid oxidation inhibitory capacities gradually increased with the increase of polyphenol content when constructing the sodium caseinate/quercetin emulsion system.

## 4 Conclusion

Alkaline-assisted processing enables the formation of CA/SPI conjugates, as evidenced by structural modifications detected through UV, FT-IR, and FS. The conjugated systems exhibited enhanced antioxidant capacity and hydrophilicity compared to native SPI, with the derived nanoemulsions demonstrating superior oxidative stability and storage performance. When employed as emulsifiers, CA/SPI conjugates generated nanoemulsions with droplet diameters below 220 nm, surpassing SPI-stabilized systems in lipid oxidation resistance during extended storage. These findings highlight the potential of polyphenol-modified protein complexes as functional emulsifiers for bioactive delivery applications in nutraceutical formulations.

## Data Availability

The raw data supporting the conclusions of this article will be made available by the authors, without undue reservation.
